# Quantitative liver proteomics identifies FGF19 targets that couple metabolism and proliferation

**DOI:** 10.1371/journal.pone.0171185

**Published:** 2017-02-08

**Authors:** Vittoria Massafra, Alexandra Milona, Harmjan R. Vos, Boudewijn M. T. Burgering, Saskia W. C. van Mil

**Affiliations:** Center for Molecular Medicine, UMC Utrecht, Utrecht, The Netherlands; University of Navarra School of Medicine and Center for Applied Medical Research (CIMA), SPAIN

## Abstract

Fibroblast growth factor 19 (FGF19) is a gut-derived peptide hormone that is produced following activation of Farnesoid X Receptor (FXR). FGF19 is secreted and signals to the liver, where it contributes to the homeostasis of bile acid (BA), lipid and carbohydrate metabolism. FGF19 is a promising therapeutic target for the metabolic syndrome and cholestatic diseases, but enthusiasm for its use has been tempered by FGF19-mediated induction of proliferation and hepatocellular carcinoma. To inform future rational design of FGF19-variants, we have conducted temporal quantitative proteomic and gene expression analyses to identify FGF19-targets related to metabolism and proliferation. Mice were fasted for 16 hours, and injected with human FGF19 (1 mg/kg body weight) or vehicle. Liver protein extracts (containing “light” lysine) were mixed 1:1 with a spike-in protein extract from ^13^C_6_-lysine metabolically labelled mouse liver (containing “heavy” lysine) and analysed by LC-MS/MS. Our analyses provide a resource of FGF19 target proteins in the liver. 189 proteins were upregulated (≥ 1.5 folds) and 73 proteins were downregulated (≤ -1.5 folds) by FGF19. FGF19 treatment decreased the expression of proteins involved in fatty acid (FA) synthesis, i.e., Fabp5, Scd1, and Acsl3 and increased the expression of Acox1, involved in FA oxidation. As expected, FGF19 increased the expression of proteins known to drive proliferation (i.e., Tgfbi, Vcam1, Anxa2 and Hdlbp). Importantly, many of the FGF19 targets (i.e., Pdk4, Apoa4, Fas and Stat3) have a dual function in both metabolism and cell proliferation. Therefore, our findings challenge the development of FGF19-variants that fully uncouple metabolic benefit from mitogenic potential.

## Introduction

Fibroblast growth factors (FGFs) are secreted signalling proteins with a wide range of functions in metabolic regulation, cell growth and differentiation, angiogenesis, embryonic development, as well as wound healing and repair [1]. Endocrine FGFs, i.e. FGF19, FGF21 and FGF23 constitute a subfamily of FGFs secreted in the circulation with roles in bile acid (BA), glucose and lipid metabolism (FGF19), metabolic adaptation during fasting (FGF21), and modulation of vitamin D and phosphate homeostasis (FGF23) [2].

FGF19 (FGF15 in rodents) is a postprandial enterokine induced by the nuclear hormone receptor Farnesoid X Receptor (FXR; NR1H4) upon activation by BAs [3]. FGF19 signals from intestine to liver via binding to FGFR4/β-klotho receptor complex to repress the gene encoding cholesterol 7α-hydroxylase (CYP7A1), which catalyses the first and rate-limiting step in the classical BA synthetic pathway [4]. In addition, FGF19 prevents lipid and glucose accumulation in the liver by inducing fatty acid oxidation and decreasing expression of acetyl coenzyme A carboxylase 2 (Acc2) involved in FA synthesis [5, 6]. Furthermore, FGF19 inhibits lipogenesis by counteracting the insulin-induced increase of sterol regulatory element-binding protein-1c (SREBP-1c) expression, a key transcriptional activator of genes involved in lipogenesis [7, 8]. FGF19 regulation of glucose metabolism involves stimulation of glycogen synthesis [9] and inhibition of gluconeogenesis via inactivation of cAMP regulatory element-binding protein (CREB) and subsequent decrease in proliferator-activated receptor g coactivator-1α (PGC-1α) [10].

The beneficial impact of FGF19 on lipid, glucose and BA homeostasis raise the possibility to pursue FGF19 as a therapeutic target for diabetes, metabolic syndrome and cholestatic liver diseases. However, the development of FGF19-based therapeutics is hampered by the mitogenic potential of FGF19 and its subsequent tumorigenic implications. FGF19-transgenic mice display increased hepatocyte proliferation at 2–4 months of age and develop hepatocellular carcinoma (HCC) at 10–12 months [11]. In concurrence with this, tumour progression in HCC patients is associated with increased FGF19 expression [12], and FGF19 gene has been shown to be a driver gene for HCC [13]. In an effort to eliminate the tumorigenic activity of FGF19 without compromising its beneficial metabolic effects, variants of FGF19 with diminished proliferative potential have been engineered, for example by eliminating the binding site to FGFR4 [14–16]. Although these results are very promising, caution should be taken since changes in metabolism have been recognized to play a driver role in oncogenesis with the ability to control both genetic and epigenetic events in cells (reviewed in [17]). It is therefore possible that the effects of FGF19 on proliferation and tumorigenesis may also be induced by its effects on metabolism.

Therefore, a comprehensive understanding of the FGF19 signalling cascade, together with mechanistic insights into the effects of FGF19 on metabolism and proliferation, are essential for the design of an FGF19-based therapeutic. Here we investigate the proteome-wide changes induced in mice upon administration of human recombinant FGF19. By using an untargeted proteomics approach, we expand the knowledge on FGF19-mediated protein expression changes and reveal that FGF19 indeed acts as a regulator of BA, lipid, glucose, amino acid metabolism and as a signalling molecule inducing expression of proliferative and tumorigenic proteins. We also show by pathway analyses that many of the proteins regulated by FGF19 function both in metabolism and proliferation, emphasizing that FGF19-mediated effects on proliferation may not so easily be eliminated without also affecting the beneficial effects on metabolism.

## Materials and methods

### Animal experiments

Wt C5Bl/6 male mice (8 weeks) were housed in groups, with enriched bedding, were fed standard chow *ad libitum*. They were fasted for 16 hours prior to treatment to ensure low endogenous FGF15 signalling. Mice received a single intraperitoneal dose of human recombinant FGF19 (1 mg/kg body weight, R&D Systems, Minneapolis, U.S.) in 0.1% saline solution or vehicle. Mice were terminated after 0 min, 15 min, 1 h, 2 h, 4 h or 12 h and liver tissue was snap frozen for RNA and protein analyses. Mice were euthanized by decapitation. This method of euthanasia was selected based on scientific need as it is the quickest, minimizes the distress and anxiety experienced by the animal, prevents stress induced pathways induced by CO_2_ inhalation and prevents glucose and other metabolic changes induced by anaesthesia. Injections and decapitations were performed by experienced staff to prevent animal discomfort. The study protocol was approved by the University Medical Center Utrecht Ethical Committee for Animal Experimentation.

### Mass spectrometry sample preparation

Liver protein extracts were generated by homogenizing 50 mg liver tissue in PBS and subsequent lysis in lysis buffer (1% NP40, 150 mM NaCl, 1 mM DTT, 50 mM Tris pH 8.0, Roche Proteinase inhibitors). Next, 100 μg protein extract from Wt or FXR-/- mice (‘light’) were mixed 1:1 with a spike-in protein extract generated from ^13^C_6_-lysine metabolically labelled mouse liver (‘heavy’) (Silantes, Munich, Germany). Proteins were denatured in urea, alkylated with iodoacetamide (Sigma, S Louis, MO, U.S.) and digested with 1 μg of trypsin (Promega, Fitchburg, WI, U.S.) using a Filtered Aided Sample Purification Protocol (FASP [18]). After trypsinization, peptides were fractionated based on their pH using Strong Anionic Exchange Chromatography and finally desalted and acidified on a C-18 cartridge (3M, St. Paul, MN, U.S.). C18-stagetips were activated with methanol, washed with buffer containing 0.5% formic acid in 80% ACN (buffer B) and then with 0.5% formic acid (buffer A). After loading of the digested sample, stage-tips were washed with buffer A and peptides were eluted with buffer B, dried in a SpeedVac, and dissolved in buffer A.

### Mass spectrometry and data analysis

Peptides were separated in a 30 cm column (75 μm ID fused silica capillary with emitter tip (New Objective)) packed with 3 μm aquapur gold C-18 material (dr Maisch, Ammerbuch-Entringen, Germany) using a 140 minute gradient (7% to 80% ACN, 0.1% FA), and delivered by an easy-nLC 1000 (Thermo, Waltham, MA, U.S). Peptides were electro-sprayed directly into an Orbitrap Fusion Tribrid Mass Spectrometer (Thermo Scientific) and analysed in Top Speed data-dependent mode with the resolution of the full scan set at 240000 and an intensity threshold of 5000 ions. Most intense ions were isolated by the quadrupole and fragmented with a HCD collision energy of 30%. The maximum injection time of the iontrap was set to 35 milliseconds.

Raw files were analysed with the Maxquant software version 1.5.1.0. [19] For identification, the mouse Uniprot 2012 was searched with both the peptide as well as the protein false discovery rate set to 1%. The SILAC quantification algorithm was used in combination with the ‘match between runs’ tool (option set at two minutes), the IBAQ and the LFQ algorithm [20]. Proteins identified were filtered for reverse hits, decoy hits and standard contaminants by using the Perseus software 1.5.1.6 [21]. The liver proteomic profile of three mice per group was determined. Light/heavy normalized ratios were used to quantify protein expression and were further processed for comparative analysis of differential expression among the experimental groups. Proteins were filtered to have more than 1 unique or razor peptide and at least two valid values per group. The mass spectrometry proteomics data have been deposited to the ProteomeXchange Consortium via the PRIDE [22] partner repository with the dataset identifier PXD005659. Analysed data are presented in the S1 File. Pathway analysis was performed using Ingenuity Pathway Analysis Program (IPA; Ingenuity Systems, Redwood City, CA, U.S.).

### Gene expression analyses

RNA was isolated from liver using TRIzol reagent (Invitrogen, Waltham, MA, U.S.). cDNA was generated from 1 μg of total RNA using SuperScript II Reverse Transcriptase (Invitrogen). qRT-PCR analysis was performed using SYBR green PCR master mix (Roche, Basel, Switzerland) and analysed on a MyIQ real time PCR cycler (BioRad, Hercules, California, U.S.). Data are presented as relative expression normalized to Gapdh gene expression. Primer sequences are listed in S1 Table.

### Western blotting

Liver tissue was homogenized in Lysis Buffer (1% NP40, 50 mM Tris HCl pH 7.4, 1 mM EDTA, 150 mM NaCl, 5 mM NaF, 0.25% sodium deoxycholate, 2 mM NaVO_3_ and protease inhibitors) using a Tissue Lyzer II (Qiagen, Venlo, The Netherlands) and protein concentration was assessed (BCA assay kit, Thermo Scientific). Western blots were probed with antibodies against Stat3 (Cat. Nr. 9139, Cell Signalling Technology, Danvers, MA, U.S.). and phospho-Stat3 (Tyr 705) (Cat. Nr. 9131, Cell Signalling Technology). α-Tubulin (Sigma), antibody was used as a loading control.

### Statistics

For the proteomic analysis a T-test was applied to determine significant differential expressed proteins between the groups (p-value <0.05). Statistical significance of pathway enrichment and upstream regulator analyses were assessed by using IPA software. Only pathways significantly enriched (setting p <0.01) are shown. For the upstream regulator analysis, p‐value measures whether there is a statistically significant overlap between the dataset genes and the genes that are regulated by a transcription factor/hormone/compound, based on the published data included in Ingenuity database. It is calculated using Fisher’s Exact Test, and significance was attributed to p‐values < 0.01.

## Results

### FGF19-mediated regulation of liver protein expression resolved by quantitative proteomics

**Fig 1:**
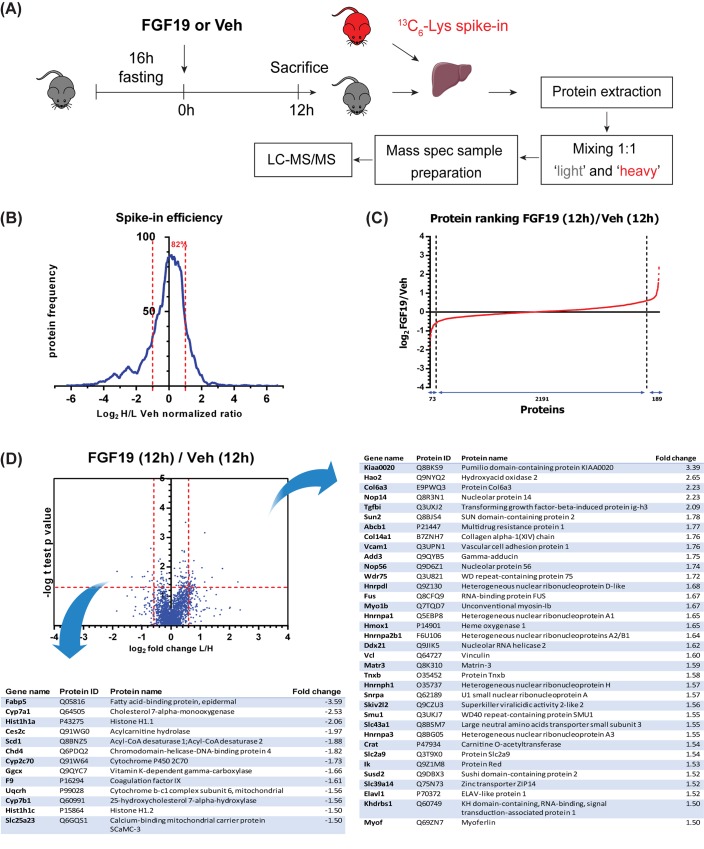
FGF19-mediated regulation of liver protein expression resolved by quantitative proteomics. (A) Schematic representation of the experimental outline to determine the hepatic proteomic profile of mice treated with FGF19 or Veh for 12 h. (n = 3) (B) Frequency plot of proteins identified in vehicle-treated Wt mice based on their total log2 heavy/light normalized ratio. The plot is representative of mean Wt untreated condition to show the basal efficiency of the heavy spike-in added to the light samples. Percentage of proteins with a log2 heavy/light normalized ratio included in interval (-1,+1) is shown. (C) Protein ranking based on changes of the log2 light/heavy normalized ratio induced by FGF19 when comparing FGF19 treatment for 12h to vehicle control. Number of proteins, of which expression was decreased (≤ -1.5 fold), unchanged or increased (≥1.5 fold) are indicated. (C) Volcano plot depicting the protein changes induced by FGF19 after 12h treatment. Plots are accompanied by tables listing the significant upregulated or downregulated proteins with fold change >1.5 (n = 3; p<0.05).

In order to characterize the metabolic and proliferative effects elicited by FGF19, we quantified protein expression changes in liver extracts from wild type mice treated with FGF19 or vehicle for 12h. Prior to FGF19 injection, mice were fasted for 16 hours, in order to reduce enterohepatic BA circulation and subsequent endogenous FGF15 signalling. Liver protein extracts (containing ‘light’ lysine) were mixed 1:1 with a spike-in protein extract from ^13^C_6_-lysine metabolically labelled mouse liver (containing ‘heavy’ lysine) and analysed by LC-MS/MS (Fig 1A). Spike-in efficiency, indicating the quality of the heavy signal as internal standard, was assessed as frequency of proteins ranked based on their log2 heavy/light normalized ratio (Fig 1B). More than 80% of proteins from the mouse liver exposed to vehicle had a heavy/light ratio close to 1, indicating a substantial equality in protein composition of the liver from our mice and the ‘heavy’ liver tissue commercially available, thereby supporting the suitability of the heavy labelled liver as internal standard for the light samples. In a scatterplot comparing FGF19- to vehicle-treated mice, light/heavy protein ratios distribute in a cloud along the diagonal with a Pearson correlation R^2^ = 0.937 (S1 Fig).

Our proteomic analysis identified 6511 proteins, of which 5459 were identified with two or more razor or unique peptides, were not reverse hits, decoy hits or standard contaminants. All proteins were identified with a minimum false discovery rate < 0.01 (Q-value, [23]). 3 mice per group were included in the analysis and 2453 proteins had at least 2 valid values in each group. No imputation of missing values by normal distribution was performed. FGF19 treatment for 12 hours resulted in upregulation (≥ 1.5 fold) of 189 proteins and downregulation (≤ -1.5 fold) of 73 proteins compared to vehicle treatment for 12 hours (Fig 1C).

Significant expression differences upon 12h FGF19 treatment are depicted in a Volcano Plot (Fig 1D). Proteins involved in cell proliferation (e.g. transforming growth factor beta-induced protein ig-h3, Tgfbi; myoferlin), metabolism (e.g. Hao2, Crat2, Abcb1), anchoring to nuclear membrane (Sun2) and nucleolar proteins (Nop14, Nop56) were among the most upregulated proteins 12h after FGF19 injection. Proteins involved in BA synthesis (Cyp7a1, Cyp7b1), lipid metabolism (Fabp5, Scd1, Ces2c), oxidative phosphorylation (Slc25a23, Uqcrh) and other metabolic processes (Ggcx, Cyp2c70) were significantly downregulated (Fig 1D). Together, the *in vivo* proteome dataset identifies FGF19 as a regulator of metabolism and proliferation, and next to yet unknown targets, identifies Cyp7A1 amongst the highest regulated genes, as was previously reported [4].

### FGF19 modulates expression of proteins involved in metabolism and cell survival

**Fig 2:**
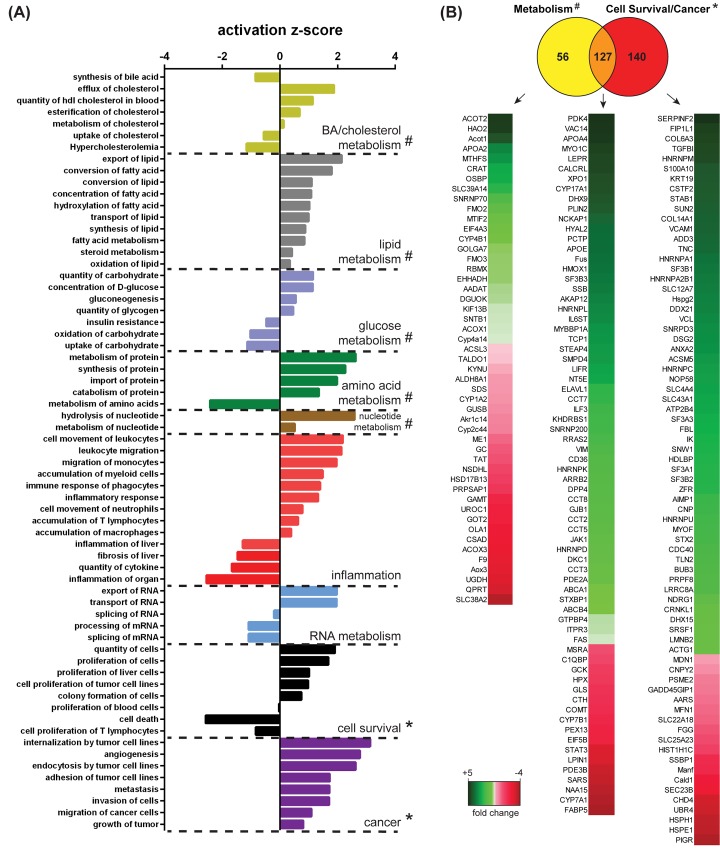
FGF19 modulates expression of proteins involved in metabolism and cell survival. (A) IPA of pathways enriched in mice treated with FGF19 for 12h compared to vehicle control. For the analysis, proteins with fold change ≥1.3 FGF19 over vehicle were included. Pathways related to physiology or disease that were significantly enriched (p-value < 0.01) are ranked in function of their activation z-score and grouped into functional classes. (B) Venn Diagram representation of proteins changed upon FGF19 treatment that are involved in metabolism and cell survival/cancer, inferred from the metabolic (#) and cell survival (*) pathways depicted in panel A. Fold change upon FGF19 treatment for proteins classified in metabolism, cell survival/cancer pathways or both is shown.

For a comprehensive understanding of FGF19 function, we performed IPA to understand which pathways were significantly enriched 12h after FGF19 treatment in comparison with the vehicle control. To have a very stringent cut-off of FGF19 targets, we did not perform imputation of missing values in each triplicate, as it is sometimes done. Consequently, the calculated number of significantly changed proteins in Fig 1 was too small for pathway analyses. We have therefore included proteins with fold change ≥ 1.3 (FGF19/vehicle) in our next analyses. FGF19 treatment yielded changes in wide ranging metabolic processes, including BA, cholesterol, lipid, glucose, amino acid, nucleotide, RNA metabolism and inflammation (Fig 2A). The pathway ‘BA synthesis’ was given a negative activation z-score, associated with decreased activity of this pathway, concurrent with the previously described role for FGF19 as enterohepatic negative regulator of BA synthesis [4]. The functional categories that can be summarized under ‘cell survival’ were enriched in the proteome dataset of the FGF19 stimulated livers, with an induction of pro-proliferative proteins and a negative activation score for proteins involved in cell death. In addition, pathways involved in tumorigenesis, such as ‘invasion’ and ‘tumour growth’ were significantly enriched and activated. Therefore, the changes observed in the liver proteomic profile of the mice receiving FGF19 confirm the role of FGF19 as a metabolic regulator, but also substantiate the concern about the tumorigenicity of FGF19 administration.

We subsequently aimed to investigate which proteins underlie FGF19-mediated regulation of metabolism and cell survival/cancer in the IPAs. Together, 183 proteins changed upon FGF19 treatment that were involved in different aspects of metabolism (e.g. Acot2, Acox1 and Acsl3) and 267 in cell survival/cancer (Col6a3. Tgfbi, Vcam1, Anxa2 and Hdlbp) (Fig 2B). However, of these proteins, 127 proteins were included in both metabolism and cell survival/cancer pathways (Fig 2B). This overlap includes Pdk4, Apoa4, Apoe, Vim, Gtpbp4, Fas (upregulated) and Stat3 (downregulated). Although the IPA algorithm is based on counting associations in published data and is therefore limited, these results suggest that separation between FGF19 proliferative and metabolic functions may be more complex than was previously anticipated.

### Evaluation of changes in gene expression involved in metabolic and proliferative function of FGF19

**Fig 3:**
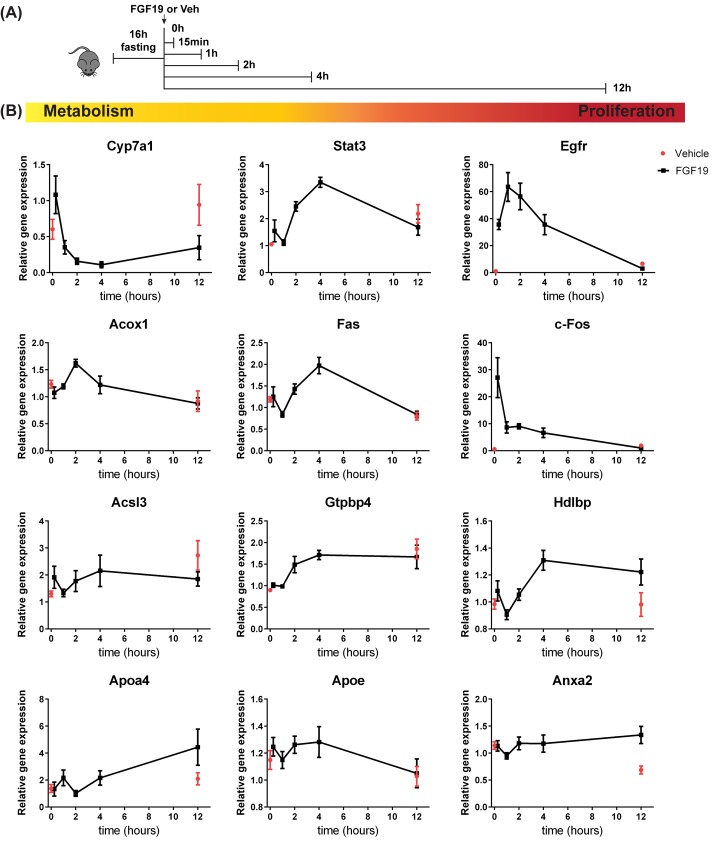
FGF19 stimulation affects mRNA expression of genes involved in metabolism and cell survival. (A) Schematic representation of the experimental outline to determine gene expression changes occurring upon FGF19 treatment for 0, 15 min, 1h, 2h, 4h and 12h. (B) Hepatic expression of genes involved in metabolism (Cyp7a1, Acox1, Acsl3), proliferation (Egfr, c-Fos, Hdlbp, Anxa2) or both (Stat3, Apoa4, Apoe, Fas, Gtpbp4) was determined by Real Time qPCR. (n = 5–6). Data are normalized to Gapdh expression and expressed as mean ± SEM.

Since FGF19 regulated the expression of the above described proteins after 12 hour treatment, we addressed whether transcriptional regulation of metabolic and proliferative genes by FGF19 precedes the up/downregulation observed at protein level. We injected mice with FGF19 and harvested the livers at t = 0, 15 min, 1h, 4h and 12h after a 16h pre-fasting period, in order to analyse FGF19 function without the confounding effect of endogenous FGF15 signalling (Fig 3A). We took along a group which received only the vehicle and was terminated after 12h, to be able to correct for differences due to prolonged starvation. Expression of the BA synthesis enzyme Cyp7a1 decreased upon FGF19 treatment, as expected (Fig 3B). The signal transducer and activator of transcription 3 (Stat3) had been previously reported to exhibit an increased phosphorylation and activation in response to FGF19, with subsequent effects both on proliferation and inhibition of FA synthesis [7, 16]. Here we show that FGF19 increases gene expression of Stat3 (Fig 3B). The mRNA expression of the proliferative markers Egfr and c-Fos (not detected at protein level in our proteome dataset) peaked within 4h after FGF19 administration, in line with a previous study [14].

We next investigated whether protein expression changes of newly identified FGF19 targets listed in Fig 2B reflect also mRNA expression regulation. Indeed, FA oxidation enzyme Acox1 mRNA expression increased and peaked 1h after FGF19 injection, whereas FA synthesis enzyme long-chain-fatty-acid-CoA ligase 3 (Acsl3) was decreased in FGF19-treated mice compared to vehicle controls (Fig 3B). Apolipoprotein Apoa4 mRNA expression increased and peaked 12h after FGF19 treatment, whereas Apoe mRNA expression was increased up to 4h after injection and was back to normal after 12h. mRNA expression of tumor necrosis factor receptor superfamily member 6 (Fas) and nucleolar GTP-binding protein 1 (Gtpbp4), implicated in both metabolism and cell survival, peaked 4h after FGF19 treatment, thus preceding the increase observed at protein level at 12h. mRNA expression of vigilin (Hdlbp), and calcium-dependent phospholipid binding protein Annexin A2 (Anxa2), both involved in cell proliferation, were increased 12h after FGF19 treatment. The change in mRNA expression of the aforementioned novel targets concur with the up/down regulation observed at protein level (Fig 2B). In contrast, Fabp5, implicated in lipid metabolism, and Tgfbi, implicated in cell survival, were not regulated at mRNA level by FGF19 (data not shown), despite their protein expression being significantly changed 12h after FGF19 injection (Fig 1D). Therefore, these gene expression studies confirm most novel metabolic and proliferative targets of FGF19, but also indicate that not all protein expression changes observed upon 12h FGF19 treatment, are preceded by a consistent change at mRNA level.

**Fig 4:**
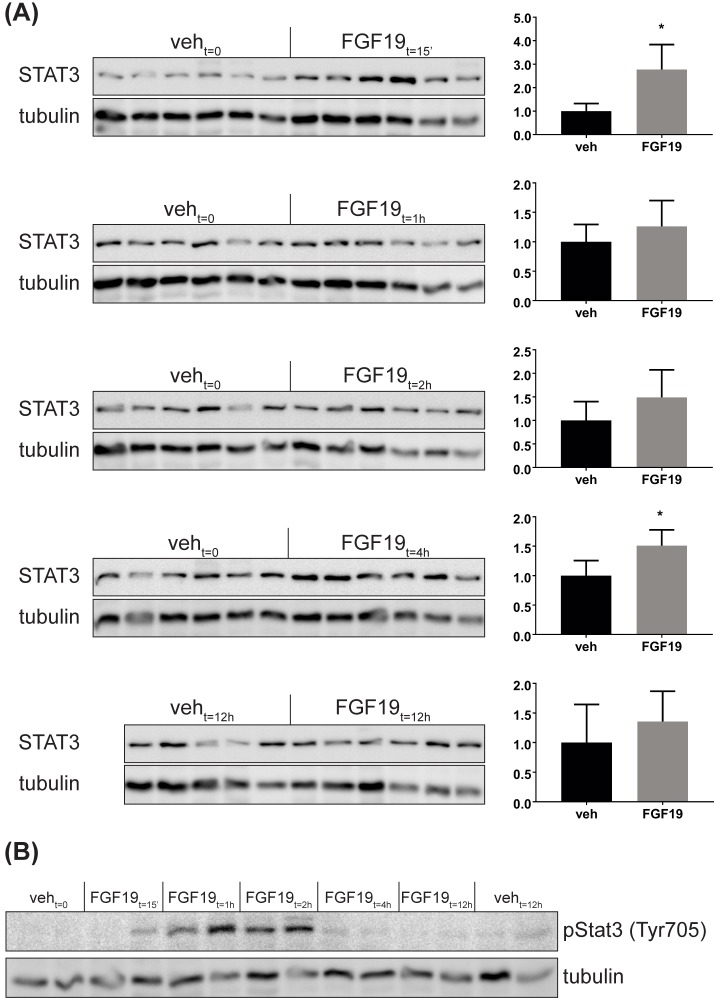
FGF19 regulates Stat3 expression and phosphorylation at Tyr705. Western blot analyses of Stat3 protein (A) and phospho-Stat3 (Tyr705) (B) at 0h, 15 min, 1h, 2h and 12h after FGF19 treatment. Quantification for Stat3 is shown as relative protein signal normalized to tubulin. Data are expressed as mean ± SD. Each lane represents one mouse liver.

In view of the high relevance of the Stat3 protein for liver homeostasis, we further analysed protein levels of Stat3 at 15 min, 1h, 2h, 4h and 12h after FGF19 treatment by Western Blotting. Stat3 protein levels were upregulated significantly at 15 min and 4 hours after FGF19 treatment (Fig 4A), in line with the gene expression data (Fig 3B). Protein levels were not significantly changed at 1, 2 and 12 hours after FGF19 treatment. Variability among the mice at these time points and lower sensitivity of the western blotting compared to the mass-spectrometry quantification may underlie the difference in the results obtained with western blot and the proteome, where we detected a small decrease in Stat3 at 12h (Fig 2B). Of note, phosphorylation of Stat3 at Tyr705 was increased at 15 min, 1h and 2h after FGF19 treatment (Fig 4B). Since phosphorylation prevents Stat3 degradation via the ubiquitin-proteasome pathway [24] and promotes nucleus translocation and activation of Stat proteins [25], it may be that the increase in Stat3 protein 15 minutes after FGF19 treatment reflects an increase in protein stability, rather than a direct transcriptional effect.

### FGF19 elicits expression changes in target genes of tumorigenic regulators

**Fig 5:**
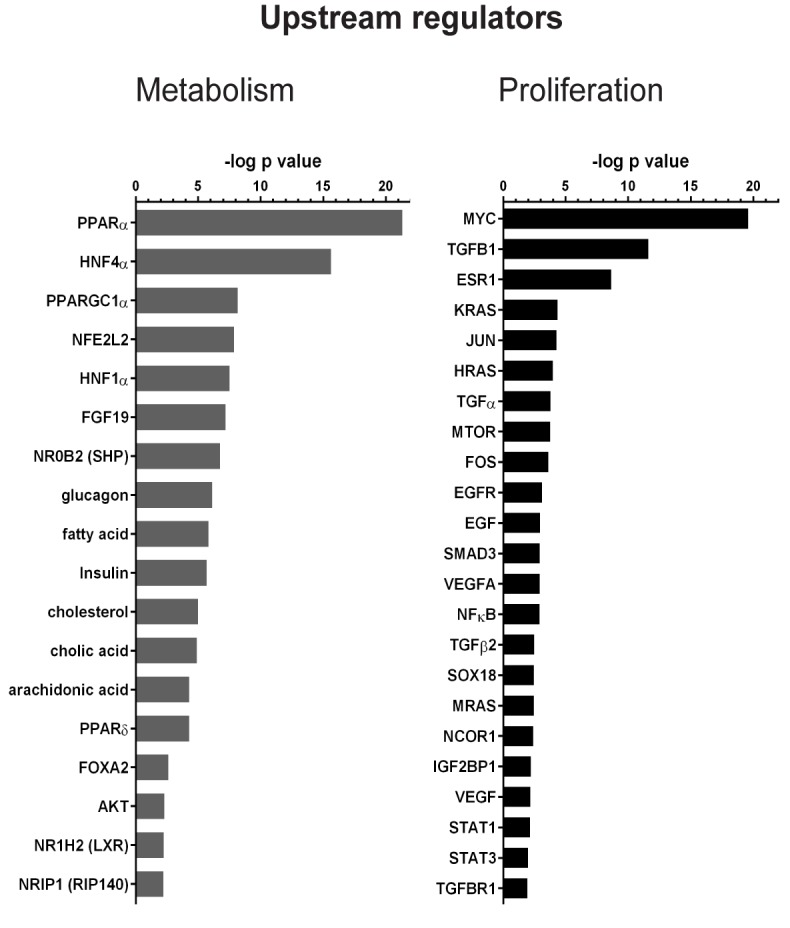
FGF19 elicits expression changes in target genes of tumorigenic regulators. (A) Ingenuity upstream regulator analysis applied to protein changes observed upon FGF19 treatment for 12h. Prediction of upstream regulators is based on the overlap between the dataset proteins and the genes that are regulated by a transcription factor/hormone/compound, based on the knowledge included in Ingenuity database (overlap p-value <0.01). Green bars, upstream regulators with positive activation z-score; grey bars, upstream regulators with negative activation z-score.

Next, we applied the Ingenuity upstream regulator analysis to the liver proteome dataset, which allows prediction of upstream regulators associated with the detected protein expression changes. We distinguished regulators of metabolism and proliferation (Fig 5). The analysis correctly identifies FGF19 and cholic acid (bile acid) as upstream regulators of the protein expression changes observed in our proteome dataset. In addition, the analysis identifies as upstream regulators the known FGF19 targets Egfr, c-Fos and the Stat3, the latter being activated by FGF19 and here reported to be regulated also at expression level. Furthermore, overlap with nuclear receptor signalling (PPARα, PPARδ, HNF4α, SHP, and LXR, PGC1α, and RIP140), transcription (NFE2L2, HNF1α, FOXA2) and insulin and glucagon was identified. In accordance with FGF19-dependent regulation of lipid metabolism, also arachidonic acid, fatty acid, and cholesterol were identified as possible upstream regulators.

Interestingly, Ingenuity analysis suggests that FGF19 may have a similar activation program as that triggered by growth factors (Vegf and Egf), oncogenes (Myc, Kras) and Tgfβ signalling (Tgfb1, Smad3, Tgfb2, Tgfbr1; Fig 4A, right panel). Tgfβ is considered both a tumor suppressor and pro-oncogenic factor [26]. FGF19 injection changed the expression of many proteins known to be regulated by Tgfβ, including Col6a3, Tgfbi, vascular cell adhesion protein 1 (Vcam1), heme oxygenase 1 (Hmox1), vinculin (Vcl) and vimentin (Vim) (Table 1). Taken together, these analyses identify similarities between FGF19 and known regulators of tumorigenesis and proliferation, indicating that FGF19 may have similar targets or mediates its effects via these regulators.

**Table 1:** TGFB1 targets regulated by FGF19.

TGFB target in the proteome dataset	FC upon FGF19 treatment	p-value	IPA Gene regulation by TGFB	Reference
**COL6A3**	2.23	0.001	Up	[27, 28]
**TGFBI**	2.09	0.011	Up	[29–31]
**VCAM1**	1.76	0.040	Down	[32, 33]
**HMOX1**	1.65	0.023	Up	[34–38]
**VCL**	1.60	0.032	Up	[39]
**VIM**	1.49	0.032	Up	[31, 40–43]
**BSG**	1.44	0.033	Up	[44]
**ITGA1**	1.44	0.032	Up	[45, 46]
**GNAI2**	1.43	0.034	Down	[47]
**ABCA1**	1.40	0.006	Up	[48–50]
**JUP**	1.37	0.024	Down	[46, 51, 52]
**DES**	1.34	0.006	Up	[53]
**PTGS1**	1.33	0.024	Up	[29, 54]

Top list of proteins changed in our proteome dataset that are predicted as TGFB1 targets by Ingenuity upstream regulator analysis. The fold change observed in our dataset upon FGF19 treatment and the direction of gene regulation (up/down) by TGFB1 inferred from literature are reported.

## Discussion

The elucidation of the molecular basis for FGF19 function is of great interest for the design of an FGF19-based therapeutic deprived of its tumorigenic potential, but retaining its beneficial effects on BA, lipid and glucose homeostasis. Investigation of the molecular mechanisms underlying FGF19 function has so far relied on targeted approaches, by addressing whether FGF19 induces the activity of key signalling proteins known to be involved in metabolism and proliferation. In the present study, we have taken an unbiased approach to determine FGF19 targets that underlie metabolic and proliferative effects. We deployed untargeted quantitative proteomics to generate a comprehensive view of FGF19 function in mouse liver. Both analysis of top regulated proteins and pathway enrichment studies in our proteome dataset support the involvement of FGF19 signalling in a wide range of processes, including BA, cholesterol, lipid, glucose, amino acid, nucleotide, and RNA metabolism, as well as cell survival and tumorigenesis. As well as decreasing the expression of BA synthesis enzyme Cyp7a1, FGF19 decreases the protein expression of Acsl3, and Scd1, implicated in FA synthesis and Fabp5, involved in FA transport. In addition, protein expression of Acox1, involved in FA oxidation and the apolipoproteins Apoe and Apoa4 are upregulated by FGF19. In almost all cases, the regulation of protein expression was preceded by a change in mRNA level. Our results on FGF19-dependent activation of growth-related pathways in the liver are in agreement with previous reports, showing that Fgf15^-/-^ mice develop less hepatocellular carcinoma as compared to Fgf15^+/+^ littermates [55], and display impaired liver regeneration [56].

An important mechanism for FGF19 induction of cell proliferation is the phosphorylation and subsequent activation of Stat3 [16]. Besides, FGF19 proliferative function requires the binding to FGFR4, since FGF19-induced increase in proliferative markers is attenuated in the liver of Fgfr4 knockout mice [14]. On the basis of this information, variants of FGF19 were engineered with reduced proliferative potential. The FGF19 variant M70 harbours 3 amino acid substitutions and a 5-amino acid deletion in the N-terminus [15]. As a result, M70 fails to activate the proliferative factor Stat3 and does not promote hepatocellular carcinoma formation in mice, while retaining the ability to maintain BA homeostasis and even to ameliorate BDL- and ANIT-induced cholestasis in mice [16, 57]. Our work shows that FGF19 increases not only Stat3 phosphorylation status (and thereby its stability) at early time points, but also Stat3 expression after treatment for 4 hours. Therefore, it would be relevant to test FGF19 variants for their ability to regulate Stat3 expression, other than activity. Another FGF19 variant (FGF19v), which does not bind and activate Fgfr4, is also devoid of proliferative effects [14]. Fgfr4 seems not be required for improvement of glucose tolerance by FGF19, therefore FGF19v may effectively control glucose homeostasis [14]. FGFR4 is essential for FGF19-dependent repression of Cyp7a1 and therefore FGF19v exhibits impaired regulation of BA metabolism [14]. These FGF19 variants deprived of tumorigenic effects are very promising from a therapeutic perspective, however, their use in clinic is challenged by the limited information available regarding FGF19 metabolic and proliferative targets. For example, although in ob/ob mice serum glucose levels were significantly decreased in mice treated with both FGF19 and M70 (24 weeks), triglycerides, cholesterol and LDL and HDL were markedly increased compared to untreated ob/ob mice [16]. This indicates that caution should be taken to interpret FGF19 actions on metabolism as beneficial under all circumstances.

It is also unclear to what extent it is mechanistically possible to discriminate FGF19 metabolic and proliferative function, as changes in metabolism are known to drive tumorigenic events [17]. Our proteome analysis reveals that FGF19 upregulates the protein expression of Tgfbi, Col6a3, Vcam1, Anxa2 and Hdlbp, that are implicated in cell survival. In the case of Hdlbp and Anxa2, but not of Tgfbi, we could show that FGF19 treatment for 12 hours determined a concurrent increase in mRNA expression. Also the mRNA expression of the proliferative markers Egfr and c-Fos was upregulated by FGF19 in our experiment, as previously described [14]. We cannot exclude that some of the observed FGF19 effects could be indirect and dependent on the induced expression of other growth factors. For example, we show that FGF19 activates a gene expression program similar to that induced by Egf. Indeed, it has been previously shown that FGF19 increases expression of Egf receptor ligands such as amphiregulin, which mediate part of the proliferative effects of FGF19 [58]. Importantly, 127 of the proteins regulated by FGF19 were annotated in both metabolism and cell survival categories in the IPA analysis, e.g. Fas, Gtpbp4 and Stat3. This number is probably an underestimation of the interplay between metabolism and cell survival pathways, since this analysis relies on publicly available data. The close interlink between metabolism and proliferation is not surprising, since metabolic reprogramming is essential for cell survival. As an example, pyruvate dehydrogenase kinase 4 (Pdk4), which is upregulated by FGF19 in our experiment (Fig 2B), provides an advantage during the proliferative state of the cell by driving the accumulation of glycolytic intermediates [59].

To overcome the limits of the intimate link between metabolic and proliferative mechanisms in designing therapeutic FGF19 variants, analysis of key tumorigenic FGF19 targets with limited or no involvement in metabolism should be addressed for dissociation between metabolic and proliferative functions. Indeed, the M70-mediated adverse effects on cholesterol and triglyceride concentrations [16] are likely due to Stat3 being the transcriptional repressor of Srebp1c [7], which activates cholesterol and fatty acid biosynthesis. Therefore blocking of Stat3 activity prevents proliferation, but also dysregulates cholesterol metabolism. Our study identifies Anxa2 and Tgfbi as possible tumorigenic FGF19 targets, without no apparent function in BA, cholesterol or lipid metabolism. The proliferative activity of Anxa2 has been associated with tumour progression, since increased Anxa2 expression correlates with a more invasive phenotype and induces proliferation and invasion signalling in human breast cancer cells [60–62]. Similarly, Tgfbi, which is a protein involved in cell adhesion and cell-collagen interactions [63], has also been implicated in tumorigenesis [64]. Intriguingly, expression of Tgfβ and its target gene Ctgf was previously shown to be decreased in Fgf15^-/-^ mice compared to Fgf15^+/+^ littermates [55]. In light of these observations, it would be informative to investigate whether these proteins critically mediate FGF19-dependent tumorigenesis. And if so, whether FGF19 variants blocking the activity of these targets are devoid of tumorigenic effects and have preserved metabolic activity.

In conclusion, our untargeted liver proteome analyses show that FGF19-mediated regulation of metabolism and proliferation is complex, and involves protein expression changes relating to BAs, glucose, lipid, amino acids, together with inflammatory, proliferative and tumorigenic processes. Future studies should address the exact mechanisms by which these proteins are regulated by FGF19, to understand whether the effects on carcinogenesis can be dissociated from beneficial effects on metabolism.

## Supporting information

S1 Fig FGF19-mediated regulation of liver protein expression resolved by quantitative proteomics. (A) Scatterplot distribution of FGF19-induced protein expression changes expressed as log2 light/heavy normalized ratios. Pearson correlation between protein expressions in FGF19-treated mice and protein expressions in Veh-treated mice is shown.
(TIF)

S1 File Proteomic analysis of livers from mice treated with FGF19 or vehicle for 12 hours. List of proteins identified in the liver and their expression values expressed as light/heavy normalized ratios. Median of three mice/group is calculated and fold change FGF19/vehicle is shown.
(XLSX)

S1 Table Mouse qRT-PCR primers. (DOCX)
