# Supplementary figures and images for: Quantitative liver proteomics identifies FGF19 targets that couple metabolism and proliferation
Source: PLoS One. 2017 Feb 8;12(2):e0171185. doi: 10.1371/journal.pone.0171185 (PMC5298232; doi:10.1371/journal.pone.0171185)

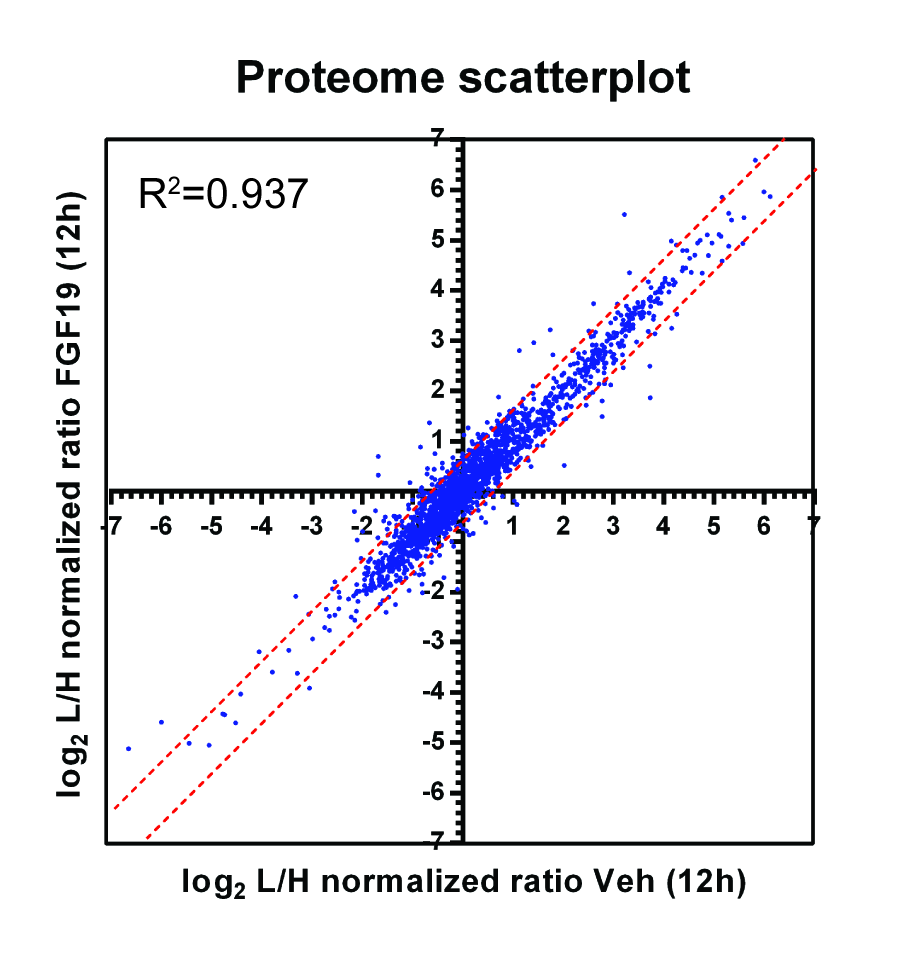

Supplement: S1 Fig — (A) Scatterplot distribution of FGF19-induced protein expression changes expressed as log2 light/heavy normalized ratios. Pearson correlation between protein expressions in FGF19-treated mice and protein expressions in Veh-treated mice is shown. (TIF) [file pone.0171185.s001.tif]
