# Supplementary material for: Quantitative liver proteomics identifies FGF19 targets that couple metabolism and proliferation
Source: PLoS One. 2017 Feb 8;12(2):e0171185. doi: 10.1371/journal.pone.0171185 (PMC5298232; doi:10.1371/journal.pone.0171185)
Supplement: S1 Table — (DOCX) [file pone.0171185.s003.docx]

**S1 Table. Mouse qRT-PCR primers**

| **Gene** | **Sequence (5’->3’)** |
| --- | --- |
| Gapdh | FW CAAGGTCATCCATGACAACTTTG |
|  | RV GGCCATCCACAGTCTTCTGG |
| Egfr | FW ACAACCCTATGAGCACCTGA |
|  | RV GAGTCGTTTGGCTGGGATAA |
| c-Fos | FW GAAGGGAACGGAATAAGATGG |
|  | RV CTGTCTCCGCTTGGAGTGTA |
| Cyp7a1 | FW ATGTCCACTTCATCACAAACTCC |
|  | RV TTTCCATCACTTGGGTCTATGC |
| Acsl3 | FW TGTCTTTCTCATGGATGCCGA |
|  | RV CAGCACGGATGTGTCTCCTT |
| Acox1 | FW CCGCCACCTTCAATCCAGAG |
|  | RV CAAGTTCTCGATTTCTCGACGG |
| Apoa4 | FW ACCCAGCTAAGCAACAATGC |
|  | RV TGTCCTGGAAGAGGGTACTGA |
| Apoe | FW CTGACAGGATGCCTAGCCG |
|  | RV CGCAGGTAATCCCAGAAGC |
| Hdlbp | FW GGAAAATGACCCTCCAACCTAC |
|  | RV GGGTACATGAAACACCTGAGTGA |
| Stat3 | FW CAATACCATTGACCTGCCGAT |
|  | RV GAGCGACTCAAACTGCCCT |
| Fas | FW AAACCAGACTTCTACTGCGATTCT |
|  | RV GGGTTCCATGTTCACACGA |
| Gtpbp4 | FW GGACGAATGTGTACTATTATCAAGAGA |
|  | RV GCGGGATAAATGTTGACGTACT |
| Anxa2 | FW ATGTCTACTGTCCACGAAATCCT |
|  | RV CGAAGTTGGTGTAGGGTTTGACT |
